# Egocentric Direction and Position Perceptions are Dissociable Based on Only Static Lane Edge Information

**DOI:** 10.3389/fpsyg.2015.01837

**Published:** 2015-11-30

**Authors:** Ryoichi Nakashima, Ritsuko Iwai, Sayako Ueda, Takatsune Kumada

**Affiliations:** ^1^RIKEN Brain Science Institute-TOYOTA Collaboration Center, RIKENWako, Japan; ^2^Graduate School of Informatics, Kyoto UniversityKyoto, Japan

**Keywords:** spatial perception, egocentric direction perception, egocentric position perception, static visual information, lane-edge information

## Abstract

When observers perceive several objects in a space, at the same time, they should effectively perceive their own position as a viewpoint. However, little is known about observers’ percepts of their own spatial location based on the visual scene information viewed from them. Previous studies indicate that two distinct visual spatial processes exist in the locomotion situation: the egocentric position perception and egocentric direction perception. Those studies examined such perceptions in information rich visual environments where much dynamic and static visual information was available. This study examined these two perceptions in information of impoverished environments, including only static lane edge information (i.e., limited information). We investigated the visual factors associated with static lane edge information that may affect these perceptions. Especially, we examined the effects of the two factors on egocentric direction and position perceptions. One is the “uprightness factor” that “far” visual information is seen at upper location than “near” visual information. The other is the “central vision factor” that observers usually look at “far” visual information using central vision (i.e., foveal vision) whereas ‘near’ visual information using peripheral vision. Experiment 1 examined the effect of the “uprightness factor” using normal and inverted road images. Experiment 2 examined the effect of the “central vision factor” using normal and transposed road images where the upper half of the normal image was presented under the lower half. Experiment 3 aimed to replicate the results of Experiments 1 and 2. Results showed that egocentric direction perception is interfered with image inversion or image transposition, whereas egocentric position perception is robust against these image transformations. That is, both “uprightness” and “central vision” factors are important for egocentric direction perception, but not for egocentric position perception. Therefore, the two visual spatial perceptions about observers’ own viewpoints are fundamentally dissociable.

## Introduction

In daily interactions with our visual-spatial environment, the perception of spatial properties is critical. Accurate spatial perception involves an ability to not only assess the attributions of objects as well as to comprehend spatial relationships among these objects (e.g., as in a layout or for relative distance between objects). This ability allows for effective spatial navigation, wayfinding, and so on (cf. Wolbers and Hegarty, 2010).

In spatial perception, the spatial location of an observer is defined as the viewpoint; many researchers have examined the effect of the viewpoint on spatial perception (e.g., Shelton and McNamara, 1997; Simons and Wang, 1998; Burgess, 2006). However, few studies have examined an individual’s perception of their own spatial location, i.e., of the viewpoint itself. Observers should always have some perception of their own spatial location relative to spatial relationships among the objects they perceive in their surroundings. Based on this assumption, the present study raises the following question: ‘*What property does the spatial perception of oneself have?*’

We named the spatial perception of oneself as “egocentric spatial perception,” which refers to the perception of direction or position of oneself based on visual information acquired in the visual field (i.e., landscape). The egocentric spatial perception is important during driving or locomotion. For example, in the driving situation, it is necessary for drivers to perceive which direction they can go along a lane as well as where they are in the lane. Related to this suggestion, previous studies of engineering models of drivers suggested that two distinct visual processes are involved in driving, particularly in steering control (Donges, 1978; Godthelp, 1986). One process involves anticipation of the direction of the car (i.e., previewing road direction), which relates to egocentric direction perception. The visual information for this process is mainly acquired at a distance relatively far from the car. The other process involves managing compensatory corrections of the car’s lateral deviation from the center of a lane, which relates to egocentric position perception. The visual information for this process is mainly acquired at a distance relatively near to the car. Consistent with this model, Land and Horwood (1995) reported that visual information from the “far” region is used to estimate road curvature whereas visual information from the “near” region is used to provide position-in-lane feedback during driving. In the present study, similar to previous studies using a driving simulator, we define the “far” and “near” regions on the basis of perspective. That is, the “far” region is the area around a vanishing point, and the “near” region is the area around the viewpoint in a 2-D pictorial plane.

In addition, recent studies have reported that, at least during locomotion, visual processes related to these two perceptions involve different neural substrates (Billington et al., 2010, 2013; Furlan et al., 2014; Huang et al., 2015). For example, the visual processing of future path information, related to egocentric direction perception, involves the posterior parietal cortex, whereas visual processing for maintenance of lateral position in the lane, related to egocentric position perception, involves MT+ (Billington et al., 2010). Further, Furlan et al. (2014) reported that the putative ventral intraparietal area (pVIP) and the cingulate sulcus visual area (CSv) strongly responded to changing heading direction.

Currently, it has been established that visual processes related to the observers’ own spatial position and direction perceptions are dissociable. However, the many previous studies that address this issue have largely examined these percepts in information-rich situations, where dynamic information (i.e., optic flow) and/or much visual information about surrounding objects can be acquired (e.g., Rushton et al., 1999; Li and Chen, 2010; Kountouriotis et al., 2012, 2013; see also Kemeny and Panerai, 2003). In such situations, it is difficult to identify what information allows observers to perceive their own perspective. The present study simplifies the visual situations presented to observers by isolating road-edge information in a dynamic context. This issue is important considering that many of the current studies on spatial perception have relied on static environments (e.g., Shelton and McNamara, 1997; Simons and Wang, 1998; Burgess, 2006).

Further, many studies examining the visual processing during driving used visuomotor control task (e.g., a steering control task conducted in a simulated driving setting). For successful visuomotor control, accurate processing is required in two stages: visual perception and motor control with the perceived visual information. To be more specific, it is necessary to perceive visual information correctly and to use the visual information appropriately for successful fine motor control. Thus, previous studies using the visuomotor control task could not provide direct evidences regarding the dissociation of two egocentric spatial perceptions themselves.

The purpose of this study was to examine the effect of the static element of road-edge information on egocentric direction and position perceptions themselves, and to clarify what factors are important for these perceptions. With respect to viewing road-edge information, there are two factors involved in viewing “far” and “near” information in the road landscape during daily situations. First, “far” visual information is seen at an upper location relative to “near” visual information (Land and Horwood, 1995; Frissen and Mars, 2014), referred to the “uprightness factor.” Second, drivers usually look at “far” visual information using central vision and “near” visual information using peripheral vision (Land and Lee, 1994; Summala et al., 1996; Salvucci and Gray, 2004), referred to the “central vision factor.” It is noted that fine visual information is processed in the central vision and coarse information is processed in the peripheral vision. This study examined how these factors influence the egocentric position and direction perceptions.

## Experiment 1

In Experiment 1, we examined whether the “uprightness factor” influences the egocentric direction and egocentric position perceptions. We prepared the two image structure conditions: a normal image condition and inverted image condition. In the inverted image condition, the normal image of a road landscape was presented in an upside-down manner, in which the road looks like a ceiling running straight. In this condition, “far” information was seen at a lower location than “near” information. If this feature is important for perception, performance would be better in the normal image condition than in the inverted image condition.

We used two detection tasks during which two images of a two-lane straight road were sequentially presented. To prevent the observers’ eye movement during image presentation, the road images were presented briefly. A relatively long blank display between the presentations of road images minimizes the effect of the persistence of vision. In the first task, referred to as the front direction detection task, observers judged which view of the road was the view when they directed to the straight ahead relative to the lane. In the second task, referred to as the center position detection task, observers judged which view of a road was the view when they located at the center of the lane. The former task corresponds with egocentric direction perception, and the latter with egocentric position perception.

### Method

#### Participants

Twenty-four young adults (mean age = 21.3 years, *SD* = 1.1 years), who were naïve to the purpose of this research, participated. All of them had normal or corrected-to-normal vision. All experiments were approved by the institutional review board of RIKEN, and written informed consent was obtained from all participants. The data from three participants who could not follow the instructions and who showed correspondingly poor task performance (around chance level) were discarded. The data from the remaining 21 participants were analyzed.

#### Stimuli

The road images were rendered from one 3-D model of a two-lane road running straight, where we simulated the natural driving scene, using Shade 11 software (e-frontier, Inc., Tokyo). The road images subtended 66.4° × 40.4° of visual angle at a viewing distance of 60 cm maintained by a forehead and chin rest. In the road images (normal road image), the vanishing point was presented at the same height as the fixation point, based on the fact that the convergence point of a converging-line image represents eye level (cf. Wu et al., 2007).

**FIGURE 1:**
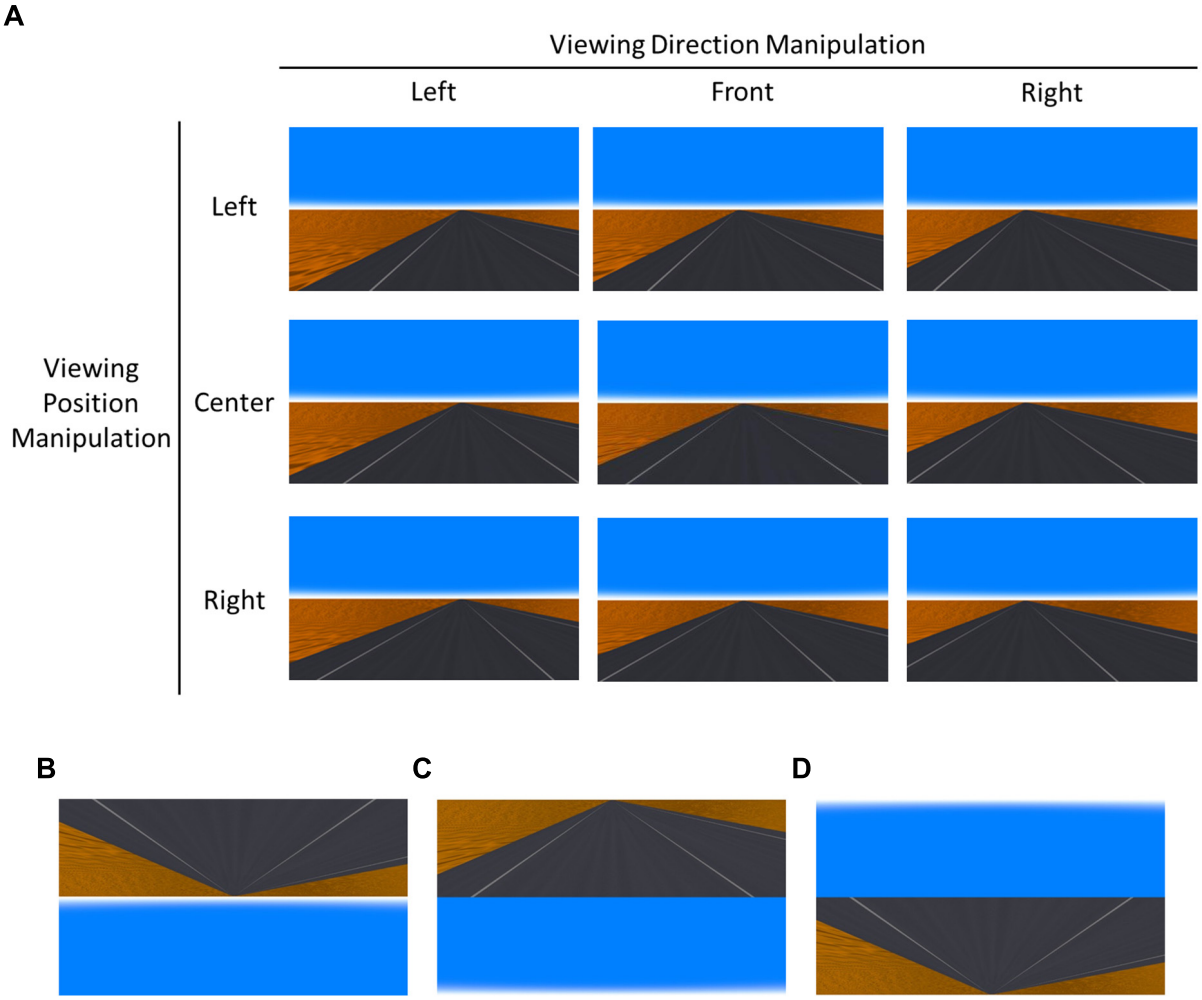
**Examples of stimuli in the experiments.** **(A)** Nine types of image in the normal image condition: three viewing direction manipulations (left, front, and right) × 3 viewing position manipulations (left, center, and right). In the front direction detection task, presented images were chosen from one row. In the center position detection task, presented images were chosen from one column. **(B)** An example of the inverted images used in Experiment 1. **(C)** An example of the transposed images used in Experiment 2. **(D)** An example of the inverted and transposed images used in Experiment 3.

For the experimental session, we created nine types of road image (see **Figure 1A**), which consisted of three viewing direction manipulations (left, front, and right) × 3 viewing position manipulations (left, center, and right). In the front viewing direction image, the road was viewed from the viewpoint whose direction was the same as that of the straight road. In the left (right) viewing direction image, the road was viewed from a viewpoint whose direction was misaligned by 2° toward the left (right) from the direction of the straight road. In the left (and right) direction images, the location of the vanishing point slightly shifted to the left (and right). In the center viewing position image, the road was viewed from the viewpoint whose position was at the center of the lane. In the left (right) viewing position image, the road was viewed from a viewpoint whose position was shifted by about 10% of the lane width toward the left (right) from the center of the lane. We named these nine images as the “normal” images. The inverted images were created by vertically flipping the normal images (**Figure 1B**).

For a practice session to familiarize participants with the task, we created four other images that comprised two viewing direction manipulations (left and right) × 2 viewing position manipulations (left and right). In the viewing direction manipulation, the road was viewed from the viewpoint whose direction was misaligned by 4° toward the left (right) from the direction of the straight road. In the viewing position manipulation, the road was viewed from the viewpoint whose position was shifted by about 20% of the lane width toward the left (right) from the center of the lane.

#### Apparatus

Presentation of the stimuli and the recordings of participants’ responses were controlled by a computer, using the Psychophysics Toolbox (Brainard, 1997; Pelli, 1997). Stimuli were displayed on a 46-inch liquid crystal display (1920 × 1080 pixels). Participants’ responses were acquired by pressing keys on a standard 10-key pad.

#### Procedure

**FIGURE 2:**
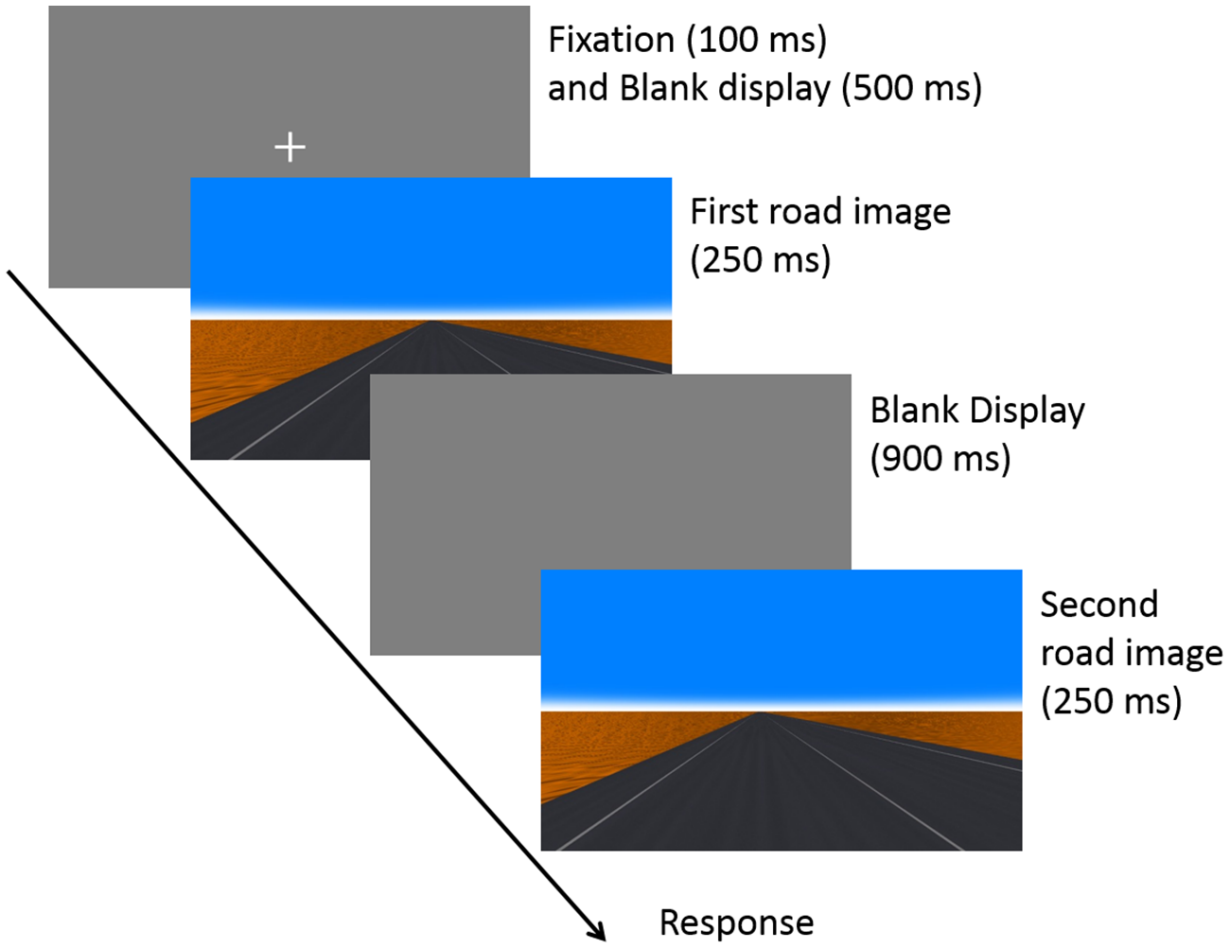
**Experimental sequence of a trial.** In both tasks, two road images were sequentially presented for 250 ms with an intervening blank display (900 ms). Participants were instructed to judge which image was viewed straight in the front direction detection task, and to judge which image was viewed from the center of the lane in the center position detection task.

Participants were seated on the chair whose position was fixed in front of the display in a dark room. Their head was fixed by a forehead and chin rest. The head horizontal position was the same as the position of the center of the display, in order to judge their actual egocentric direction and position correctly based on the road landscape. At the beginning of each trial, a fixation cross was presented and the participants were instructed to press the “5” key to start the trial when they were ready. During a trial, participants were told to gaze at the location (i.e., to look straight ahead) and not to move their eyes. They were instructed to look the picture planes as if they saw a road landscape through a windshield of a car. That is, they understood that they could not see themselves in the picture in this experiment. After their keypress, the fixation cross was visible for 100 ms and then it was replaced by a blank display presented for 500 ms. Then, two road images (each 250 ms) were presented sequentially, separated by a blank screen for 900 ms (**Figure 2**). After the presentation of the road images, a response display was presented until participants provided their responses. In the response display, the phrase “1st or 2nd?” was displayed, indicating that if observers judged the 1st image to be the correct answer, they should press the left-key (“4”), or otherwise the right-key (“6”). The correct answer was the front viewing direction image in the front direction detection task, and the center viewing position image in the center position detection task.

The front direction detection task presented two stimuli. One presented a road image viewed from a viewpoint with an orientation identical to that of the straight road. The other road image was generated from a viewpoint perspective misaligned either toward the left or toward the right from the straight road. The order of the presentation of these two stimuli was counterbalanced across trials. Participants were instructed to judge which of the two images represented a straight view, i.e., their viewing direction was the same as the road direction, by pressing one of two keys as accurately as possible. In this task, the viewing position of the road image was manipulated (viewing position condition) by increasing the variation of stimuli to avoid monotony. In a given trial, the viewing positions of the two images were identical. For example, on one trial, the two presented stimuli represented, respectively, an image where the viewing direction was front from the *left viewpoint position* in the lane and an image where viewing direction was left from the *left viewpoint position* in the lane.

In the center position detection task, a road image viewed from a viewpoint at the center of the lane was paired with a road image viewed from a viewpoint either to the left or right of the center of the lane. Participants were instructed to judge which was the image that they viewed from the center of the lane by pressing one of two keys as accurately as possible. In this task, similar to the front direction detection task, viewing direction of the road image was manipulated (viewing direction condition), again to avoid task monotony. In a given trial, the road directions of the two images were the same. For example, on one trial, two presented stimuli were, respectively, the image where the viewpoint position was center in the lane with the *left viewing direction* and the image where viewpoint position was left in the lane with the *left viewing direction*.

The participants completed an experimental session of 240 trials, with 40 trials in each of the six conditions created by the 2 (image structure) × 3 (viewing positions in the direction detection task/viewings direction in the position detection task) factorial design. Trial order was randomly determined. All participants completed both tasks, with task order being counterbalanced across participants. Before the experimental session of each task, participants completed the practice session (12 trials) to become familiarized with the task. The practice session made participants understand that it was not effective to gaze at the location other than the fixation cross, because they could not know the presented image was normal or inverted image beforehand. In the practice session, participants were given feedback for their response (i.e., correct or not). The practice session was repeated until their percentage of correct response became better than about 70%. For almost all participants, one practice session was enough.

### Results and Discussion

**FIGURE 3:**
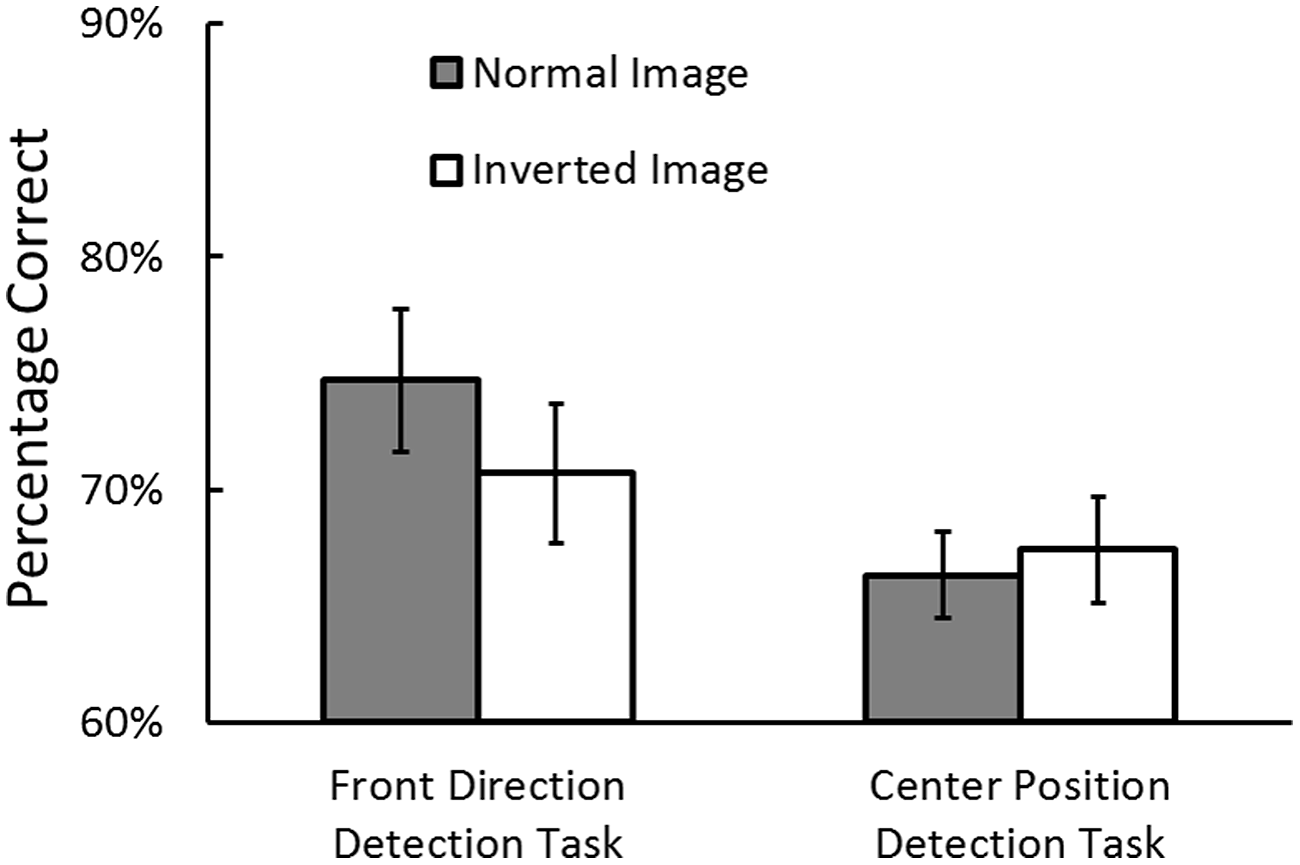
**Percentage of correct responses for the front direction detection task (egocentric direction perception) and center position detection task (egocentric position perception) in Experiment 1.** Error bars indicate standard errors.

Prior to the analyses, the trials where RTs exceeded 10 s were removed as outliers (0.28% of the trials in the direction detection task and 0.26% in the position detection task). Although we manipulated the viewing positions in the direction detection task/viewings direction in the position detection task, we analyzed the data collapsed over the conditions, because this manipulation was not our main purpose. **Figure 3** shows the percentage of correct responses in the front direction detection task (i.e., egocentric direction perception) and in the center position detection task (i.e., egocentric position perception). We conducted an analysis of variance (repeated measures ANOVA) on task performance, with the two factors of task (direction task vs. position task) and image structure (normal vs. inverted) as independent variables. The main effect of the task was marginally significant, *F*(1,20) = 3.41, *p* = 0.08, $\eta_{p}^{2}$ = 0.14, indicating that the performance was better in the direction task than in the position task. The main effect of image structure was not significant, *F*(1,20) = 1.25, *p* = 0.28, $\eta_{p}^{2}$ = 0.06. Importantly, the interaction was significant, *F*(1,20) = 5.48, *p* = 0.03, $\eta_{p}^{2}$ = 0.22. This interaction indicated that egocentric direction perception performance was better in the normal image condition than in the inverted image condition, *p* = 0.03, whereas egocentric position perception performance was not different between in the normal image condition than in the inverted image condition, *p* = 0.50.

We conducted an ANOVA on Reaction Times (RTs) in the conditions (see **Table 1**). RTs were shorter in the front direction detection task than in the center position detection task, *F*(1,20) = 4.21, *p* = 0.05, $\eta_{p}^{2}$ = 0.17. The main effect of image structure and the interaction were not significant, image structure: *F*(1,20) = 0.76, *p* = 0.39, $\eta_{p}^{2}$ = 0.04, the interaction: *F*(1,20) = 0.45, *p* = 0.50, $\eta_{p}^{2}$ = 0.02. We confirmed there was no speed–accuracy tradeoff.

**Table 1:** Summary of Reaction Times (ms) in the experiments (Mean ± SE).

	Front direction detection task		Center position detection task	
		
	Normal image	Modified image	Normal image	Modified image
Experiment 1	1275 ± 65	1225 ± 61	1421 ± 73	1411 ± 98
Experiment 2	1212 ± 45	1209 ± 34	1315 ± 57	1302 ± 58
Experiment 3	1241 ± 51	1307 ± 59	1364 ± 68	1366 ± 58


**Modified image indicates the inverted image in Experiment 1, the transposed image in Experiment 2, and the inverted and transposed image in Experiment 3.**

Egocentric direction perception performance was better in the normal image condition. In other words, performance was better for a ground-like surface than for a ceiling-like surface. This is consistent with the ground dominance effect reported in previous studies of visual perception (e.g., Rushton et al., 1999; Bian et al., 2005, 2006). The ground dominance effect refers to the phenomenon where observers show a preferred response according to the optical contact information provided by the ground surface. The advantage of ground surface information can apparently be applied to egocentric direction perception, in addition to distance perception (Bian et al., 2005, 2006; Bian and Anderson, 2011), heading (Rushton et al., 1999), vection (Sato et al., 2007), change detection (Bian and Anderson, 2010) and visual search (McCarley and He, 2000; Imura and Tomonaga, 2013). In this experiment, participants should look at “far” visual information (i.e., the vanishing point) using central vision and “near” visual information using peripheral vision in both image conditions, because the fixation point, which they were instructed to gaze at, was located close to the vanishing point. Thus, the result that performance differed between these two image conditions indicates that the “uprightness factor” (Land and Horwood, 1995; Frissen and Mars, 2014) is important for egocentric direction perception.

Egocentric position perception performance was not changed by the image inversion, indicating no advantage of ground surface for the egocentric position perception. Thus, the layout of “far” and “near” visual information is not a decisive factor for the egocentric position perception. In general, observers perceive their own position by mainly using “near” visual information which is usually acquired from the peripheral vision. Visual information acquired from the peripheral vision is coarse (i.e., low-quality), indicating that basically this perception can be accomplished by coarse visual information. Thus, image inversion does not influence the egocentric position perception.

In the driving literatures (e.g., Donges, 1978; Frissen and Mars, 2014), it has been suggested that there are two distinct visual processes for stable steering control, compensation related to egocentric position perception and anticipation corresponding to egocentric direction perception. The result of this experiment suggests that the visual perceptions themselves are dissociable in the limited visual information environment where only static road-edge information is available.

## Experiment 2

Experiment 1 indicated that the “uprightness factor” is important for egocentric direction perception. In Experiment 2, we examined the second “central vision factor.” To test this, we prepared a new image structure condition: a transposed image condition, in which the upper half of the normal image was presented under the lower half (see **Figure 1C**). In the transposed image condition, “far” information (i.e., vanishing point) was provided to the peripheral visual field and “near” information to the central visual field. If this factor is important for egocentric direction perception, performance should be better in the normal image condition than in the transposed image condition.

In addition, we examined the effect of the “central vision factor” on egocentric position perception. Egocentric position perception requires access to “near” visual information, and this information is usually relatively coarse, given that drivers look at the “near” region via their peripheral vision. We examined whether egocentric position perception is improved when the “near” region is seen via central vision, where the resolution is high.

### Method

#### Participants

Twenty-four young adults (mean age = 21.0 years, *SD* = 1.0 years), participated. All of them had normal or corrected-to-normal vision. None of them participated in Experiment 1. The data from three participants who could not follow the instructions were discarded. The data from the remaining 21 participants were analyzed.

#### Stimuli and Condition

The transposed images were created by switching the upper and lower halves of the normal images (**Figure 1C**). As Experiment 1, the participants completed an experimental session of 240 trials, with 40 trials in each of the six conditions that comprised the 2 (image structure) × 3 (viewing positions for the direction detection task/viewing directions for the position detection task) factorial design. Trial order was randomly determined.

#### Apparatus and Procedure

The apparatus and procedure were identical to those used in Experiment 1.

### Results and Discussion

**FIGURE 4:**
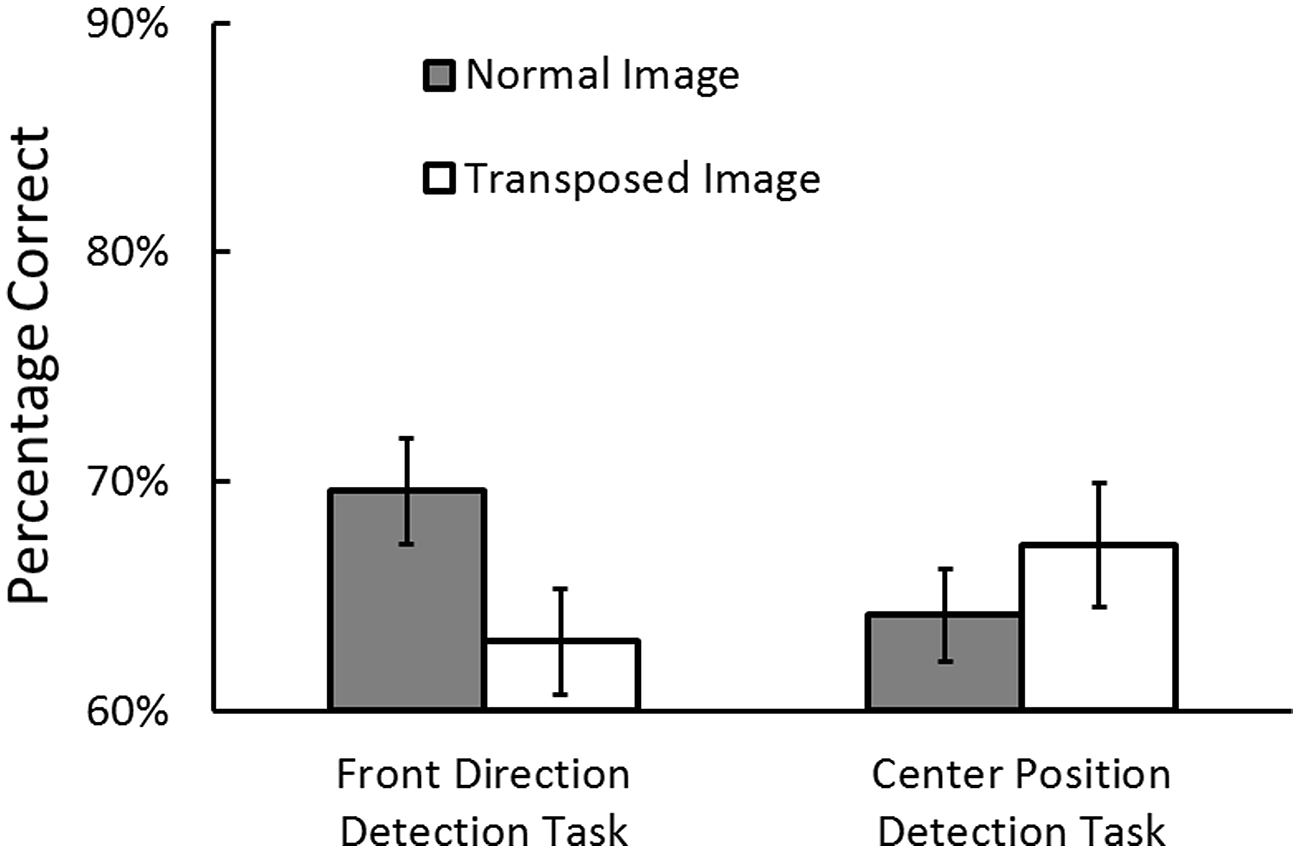
**Percentage of correct responses for the front direction detection task and center position detection task in Experiment 2.** Error bars indicate standard errors.

Prior to the analyses, the trials where RTs exceeded 10 s were removed as outliers (0.54% of the trials in the direction detection task and 0.24% in the position detection task). **Figure 4** shows the percentage of correct responses in the front direction detection task and in the center position detection task. We conducted an ANOVA on task performance with task (direction task vs. position task) and image structure (normal vs. transposed) as independent variables. The main effects of task and image structure was not significant, task: *F*(1,20) = 0.16, *p* = 0.60, image structure: *F*(1,20) = 2.96, *p* = 0.10. Importantly, the interaction was significant, *F*(1,20) = 10.88, *p* = 0.003, $\eta_{p}^{2}$ = 0.35. This interaction showed that egocentric direction perception performance was higher in the normal image condition than in the transposed image condition, *p* = 0.001, whereas egocentric position perception performance was not significantly different between two image conditions, *p* = 0.12.

We conducted an ANOVA on RTs in the conditions (see **Table 1**). RTs were shorter in the front direction detection task than in the center position detection task, *F*(1,20) = 4.34, *p* = 0.05, $\eta_{p}^{2}$ = 0.18. The main effect of image structure and the interaction were not significant, image structure: *F*(1,20) = 0.15, *p* = 0.70, $\eta_{p}^{2}$ < 0.01, the interaction: *F*(1,20) = 0.05, *p* = 0.82, $\eta_{p}^{2}$ < 0.01. We confirmed there was no speed–accuracy tradeoff.

This result indicates that the “central vision factor” is important for egocentric direction perception. This result also indicates that the visual road information presented at the upper visual field interferes with egocentric direction perception, even when such information is presented in an upright orientation (i.e., ground-like surface). This is inconsistent with the results of previous studies reporting the ground dominance effect (Bian et al., 2006; Bian and Anderson, 2010); performance can be determined mainly due to the characteristics of the ground-like surface, with the location in the visual field having little effect. This inconsistency may result from task differences. In previous studies (Bian et al., 2006; Bian and Anderson, 2010), observers were required to judge a target presented in the visual scene image, such as the relative distance judgment of targets or change detection of a target in a scene. In this study, however, the observers’ task was to judge the direction of themselves based on the road landscape. Thus, the egocentric spatial perception has different properties from the spatial perception about objects viewed by the observers.

In this experiment, the layout of the road was normal, i.e., the “far” point was seen at the upper location of the display relative to the “near” region. This indicates little (or no) advantage of looking “near” in the central visual field for egocentric position perception. Again, we show that the egocentric spatial perceptions were dissociable in the limited visual information environment.

## Experiment 3

The results of Experiments 1 and 2 lead to the following two conclusions. First, it is important to look at “far” visual information, which is presented on the upper than “near” information, using the central vision for egocentric direction perception. Second, egocentric position perception appears to be robust against manipulations of the road scene that break the appearance of a normal road landscape. However, the image structure conditions in Experiments 1 and 2 differed not only in terms of road landscape factors but also in the visual field where the road image was presented. That is, the road was presented at the lower visual field in the normal image condition and at the upper visual field in the inverted image and transposed image conditions.

In Experiment 3, we examined whether presenting the road at the upper visual field interfered with egocentric direction perception. We prepared a new image structure condition: an inverted and transposed image condition (**Figure 1D**). In this condition, the inverted road image was presented at the lower visual field, in which both “uprightness factor” and “central vision factor” were disrupted. If these factors are truly important for egocentric direction perceptions, then again, performance should be better in the normal image condition than in the inverted and transposed image condition. In contrast, if viewing the road in the lower visual field alone is important, performance should be comparable in the two image structure conditions.

In addition, we sought to confirm the robustness of egocentric position perception under this manipulation of the visual scene. We expected performance in the position detection task to be consistent across conditions.

### Method

#### Participants

Twenty-one young adults (mean age = 21.2 years, *SD* = 1.4 years) participated. All of them had normal or corrected-to-normal vision. None of them participated in Experiments 1 and 2. The data from two participants who could not follow the instructions and one participant whose data featured many outliers (15.8% of the trials) were discarded. The data from the remaining 18 participants were analyzed.

#### Stimuli and Condition

Inverted and transposed images were created by switching the upper and lower halves of the inverted images (**Figure 1D**). The participants completed an experimental session of 240 trials, with 40 trials in each of the six conditions that formed the 2 (image structure) × 3 (viewing positions in the direction detection task/viewing directions in the position detection task) factorial design. The order of trials was randomly determined.

#### Apparatus and Procedure

The apparatus and procedure were identical to those of Experiments 1 and 2.

### Results and Discussion

**FIGURE 5:**
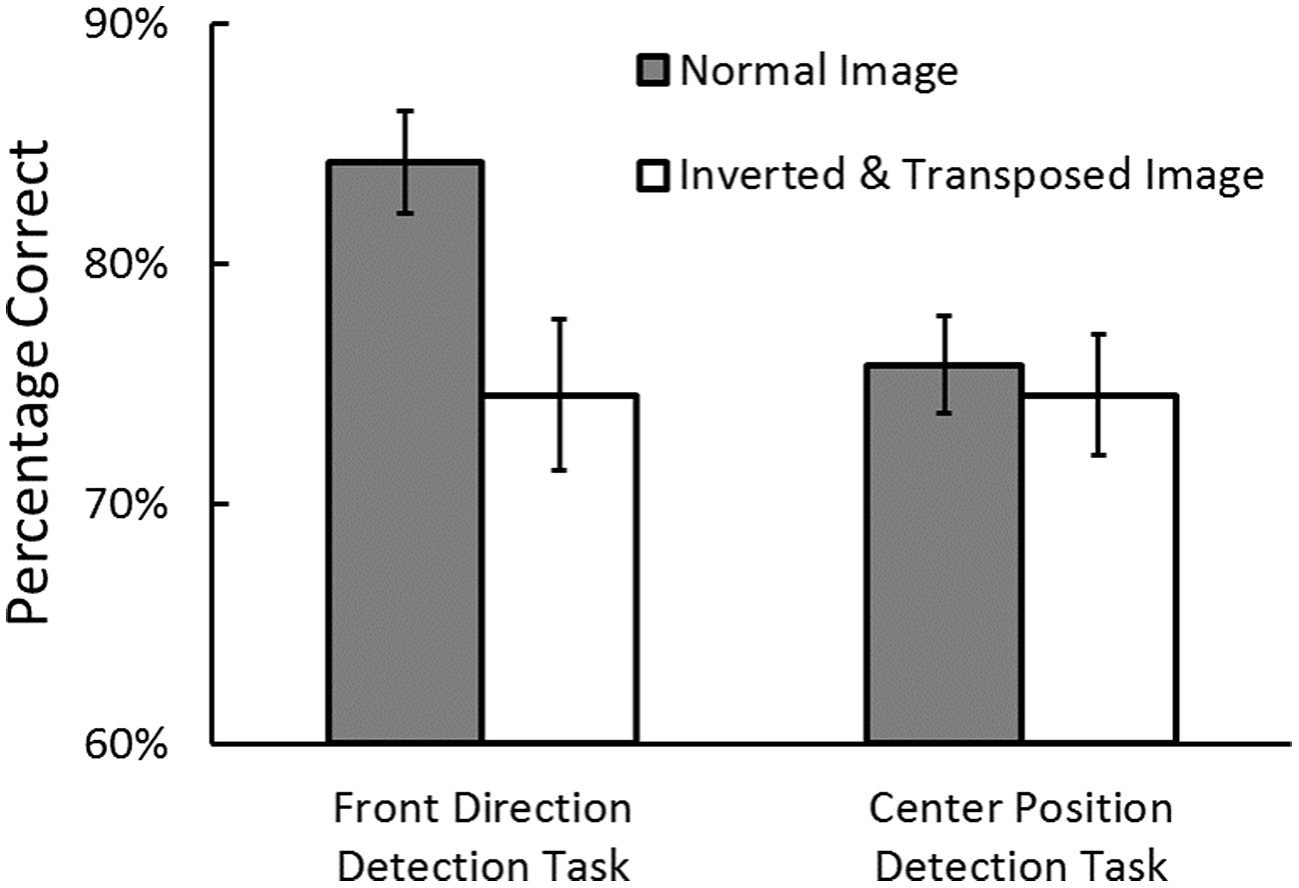
**Percentage of correct responses for the front direction detection task and center position detection task in Experiment 3.** Error bars indicate standard errors.

Prior to the analyses, the trials where RTs exceeded 10 s were removed as outliers (0.53% of the trials in the direction detection task and 0.41% in the position detection task). **Figure 5** shows the percentage of correct responses in the front direction detection task and in the center position detection task. We conducted an ANOVA on task performance with task (direction task vs. position task) and image structure (normal vs. inverted and transposed) as independent variables. The main effect of task did not reach significance, *F*(1,17) = 2.80, *p* = 0.11, $\eta_{p}^{2}$ = 0.14. The main effect of image structure was significant, *F*(1,17) = 11.87, *p* = 0.003, $\eta_{p}^{2}$ = 0.41, showing that the perception performance was higher in the normal image condition than in the inverted and transposed image condition. However, the interaction was significant, *F*(1,17) = 7.59, *p* = 0.01, $\eta_{p}^{2}$ = 0.30. This interaction showed that egocentric direction perception performance was higher in the normal image condition than in the inverted and transposed image condition, *p* < 0.001, whereas egocentric position perception performance was not different between two image conditions, *p* = 0.60.

We conducted an ANOVA on RTs in the conditions (see **Table 1**). No main effects and interaction were significant, task: *F*(1,20) = 2.34, *p* = 0.14, $\eta_{p}^{2}$ = 0.12, image structure: *F*(1,20) = 1.21, *p* = 0.29, $\eta_{p}^{2}$ = 0.07, the interaction: *F*(1,20) = 1.45, *p* = 0.24, $\eta_{p}^{2}$ = 0.08. We confirmed there was no speed–accuracy tradeoff.

This result indicates that both “uprightness factor” and “central vision factor,” rather than the viewing the road in the lower visual field factor, is important for egocentric direction perception. Taking the results of this experiment and Experiment 2 together, the ground dominance effect in egocentric direction perception appears to be due to both the characteristics of the ground-like surface and presentation at the lower visual field. In addition, the results of Experiments 1, 2, and 3 consistently show the robustness of egocentric position perception against the layout of the road image.

The results also indicates that there is little effect of the location of road presented in the visual field on the egocentric spatial perception. Previous studies suggested the different functions of the upper and lower visual fields (e.g., Previc, 1990; Brown et al., 2005). The lower visual field is specialized for the peripersonal space and the upper visual field is for the extrapersonal space. If this suggestion is always true, the egocentric direction perception performance could be better in the inverted or transposed image condition than in the normal image condition, because the direction perception requires mainly “far” visual information, i.e., visual information in the extrapersonal space. Also, the egocentric position perception performance could be better in the normal image condition than in the inverted or transposed image condition. However, our results are inconsistent with these suggestions. Thus, the results in this study suggest the special characteristics of egocentric spatial perception which are different from those of spatial perception revealed previously.

## General Discussion

Spatial perception is very important to interact with our visual environment. When we perceive visual objects in our external environment, at the same time, we should perceive ourselves in the environment as a viewpoint. However, little is known about egocentric spatial perception, e.g., the perception of the position of ourselves in the environment, especially spatial perception in a local visual environment. Some previous studies have suggested that two distinct visual processes, namely anticipation, related to egocentric direction perception, and compensation, related to egocentric position perception, are involved in driving (Donges, 1978; Godthelp, 1986). These studies examined visual process related to the egocentric spatial perception within a rich visual information environment where many object information and dynamic information can be used. Thus, it is not clear what property determines such egocentric spatial perceptions in a limited visual information environment, because many factors influencing the perceptions existed in the previous studies. In addition, the suggestions of previous studies are based on visuomotor control task findings. Since visuomotor control contained visual perception and motor control with the perceived visual information, it has been unclear the properties of the egocentric perceptions themselves.

This study focused on two types of egocentric spatial perception, egocentric direction perception and egocentric position perception, and examined the properties of these perceptions in the limited visual information environment where only static road-edge information was available. The effects of two critical visual factors of a normal road landscape on these perceptions were investigated. Through the experiments, we showed that these two perceptions themselves have different properties.

Experiment 1 showed that egocentric direction perception performance was higher when “far” visual information was seen at an upper position relative to “near” visual information than the information was arranged in the vertically inverted layout (the “uprightness factor”). This result supports the ground dominance effect in egocentric direction perception. Experiment 2 showed that egocentric direction perception performance was higher when “far” information was seen in the central vision than in the peripheral vision (the “central vision factor”). Experiment 3 confirmed that both of these factors are important for egocentric direction perception. Although we especially focused on the effect of these two factors, many other types of the factors (i.e., image disruption) can interfere with the egocentric direction perception. It is important to examine the generalizability of our results in the future research.

In order to examine the impact of two factors on egocentric direction perception, we calculated the ratio of the difference between the performance in the manipulated image condition and the normal image condition to the performance in the normal image condition. The mean ratios of performance impairment by image manipulation were subjected to a one-way ANOVA with experiment as a between-participants factor. Although the differences were only marginally significant, *F*(2,57) = 2.31, *p* = 0.10, $\eta_{p}^{2}$ = 0.08, the mean ratios of performance impairment by image manipulation in Experiment 3 (11.7 ± 2.6%; Mean ± SE) was larger than those in Experiment 2 (9.1 ± 2.2%) and Experiment 1 (4.7 ± 2.2%) for the direction detection task. This corresponds to the number of disruptions of visual factors on a road landscape. One of the features of a road landscape was disrupted in Experiments 1 and 2, and both of them were disrupted in Experiment 3. This result suggests that the both factors of a road landscape are critical for egocentric direction perception. Furthermore, this result also implies that looking at “far” information (e.g., a vanishing point) via central vision is very important for egocentric direction perception, because the performance impairment was larger in Experiment 2 than in Experiment 1.

The present results imply that two factors pertaining to the road landscape (i.e., the “uprightness factor” and “central vision factor”) additively influence egocentric direction perception. The “central vision factor” appears to be specific to egocentric direction perception. Many previous studies have reported that the “uprightness factor” (where the display looks like a ground surface) is important for various perceptions, which is called the ground dominance effect (e.g., Rushton et al., 1999; McCarley and He, 2000; Bian et al., 2005, 2006; Bian and Anderson, 2010, 2011). Further, some of these studies (Bian et al., 2006; Bian and Anderson, 2010) indicate that the “central vision factor” has little effect on such perceptions. On the other hand, egocentric direction perception requires observers to estimate the direction of themselves based on the visual scene image. In this case, fine “far” visual information acquired via central vision should be important. In this study, we clarify one difference of spatial perception properties between about visual target and about oneself.

In contrast, there was little effect of the two road landscape factors on egocentric position perception. Viewing “near” visual information via central vision affords little or no advantage for egocentric position perception. For this perception, since relatively coarse visual information can be sufficient (cf. Summala et al., 1996), perception accuracy could be high even when “near” information is degraded by peripheral presentation.

Overall, the performance in the front direction detection task (egocentric direction perception) was better than in the center position detection task (egocentric position perception). This result supports the suggestion that egocentric direction perception is mainly based on visual information from central vision and that egocentric position perception is mainly based on visual information derived from peripheral vision. The visual functions are different between the central vision and peripheral vision in scene viewing (Rayner, 2009). Visual information necessary for the core of visual cognition is mainly acquired from the central vision, where fine visual information is processed. Visual information acquired from peripheral vision, where coarse information is processed, is used to guide gaze control for information processing. The egocentric direction perception performance was high because fine and high-quality visual information could be used. It is noted that visual motion can be perceived better in peripheral vision than in central vision. Thus, in dynamic scene viewing like driving situation, the egocentric position perception performance can become high because the motion information can be used (cf. Turano et al., 2005). It is necessary to examine the effect of motion information, in addition to the knowledge based on our current results about static information, on the egocentric perception in the future research.

The results of this study indicate that egocentric position perception is more robust than egocentric direction perception when compared with image inversion or image transposition. This is consistent with the results of previous study examining the visual processes related to these spatial perceptions in an information rich visual environment (Frissen and Mars, 2014). Therefore, we suggest that these two percepts themselves are fundamentally dissociable rather than dissociable only when much visual information (dynamic and static information) is available. Previous studies have reported that neural areas related to these perceptions are different from those involved in locomotion where dynamic visual information is acquired (Billington et al., 2010, 2013; Furlan et al., 2014; Huang et al., 2015). We propose that neural area(s) other than those related to visual motion processing could also involve these two percepts. Further research is necessary to clarify this interesting issue.

In both tasks, mean performance was different in the normal image condition across the three experiments, although observers viewed exactly the same road image in this image structure condition. At this time, we just suggest that individual differences in perceptual abilities may have produced this difference. We intend to examine this issue in detail in future research.

## Conclusion

We report two spatial perceptions about oneself (egocentric spatial perception), egocentric direction perception and egocentric position perception in the limited visual information environment. Egocentric position perception is more robust than egocentric direction perception against image inversion or image transposition. Thus, these perceptions are fundamentally dissociable.

## Conflict of Interest Statement

The authors declare that the research was conducted in the absence of any commercial or financial relationships that could be construed as a potential conflict of interest.
